# Differential Effects of Sleep Disturbance and Malnutrition on Late-Life Depression Among Community-Dwelling Older Adults

**DOI:** 10.3389/fpsyt.2022.820427

**Published:** 2022-05-06

**Authors:** Gyubeom Hwang, Yong Hyuk Cho, Eun Jwoo Kim, Ji Won Woang, Chang Hyung Hong, Hyun Woong Roh, Sang Joon Son

**Affiliations:** ^1^Department of Psychiatry, Ajou University School of Medicine, Suwon, South Korea; ^2^Suwon Geriatric Mental Health Center, Suwon, South Korea

**Keywords:** sleep disturbance, malnutrition, late-life depression, interaction, conditional effect

## Abstract

**Background:**

Late-life depression is a complex phenomenon that cannot be fully understood simply as depression occurring in older adults, prompting researchers to suggest that it represents a component of geriatric syndrome. Given the inherent complexity and multifactorial nature of geriatric syndrome, understanding the interactions between the comorbid conditions involved is important for establishing appropriate preventive strategies. While sleep disturbance and malnutrition are common manifestations of geriatric syndrome, they have also been regarded as indicators of late-life depression. However, the differential effects of sleep disturbance and malnutrition on late-life depression and their interrelationships remain unclear.

**Objective:**

The objective of this study was to examine the effects of sleep disturbance and malnutrition on depression and the interactions between them among community-dwelling older adults.

**Methods:**

Sleep disturbance and malnutrition in 1,029 community-dwelling older adults from Suwon Geriatric Mental Health Center were assessed using the Pittsburgh Sleep Quality Index (PSQI) and Mini Nutritional Assessment (MNA), respectively. The Korean version of the Short Form of the Geriatric Depression Scale (SGDS-K) was used to evaluate depressive symptoms. Sociodemographic parameters were recorded. A multiple linear regression analysis was conducted to examine the effects of sleep and nutrition on depressive symptoms after adjusting for covariates. The effect size and conditional effects of sleep disturbance and malnutrition on late-life depression were assessed using Cohen’s f^2^ values and the Johnson–Neyman technique, respectively.

**Results:**

After possible confounders were adjusted, the SGDS-K score was positively associated with the PSQI score (standardized beta = 0.166, *P* < 0.001) and negatively associated with the MNA score (standardized beta = −0.480, *P* < 0.001). The local effect size of the associations was small for PSQI and medium for MNA. A significant interaction was observed between the PSQI and MNA scores. The result of the Johnson–Neyman technique indicated that the influence of PSQI on SGDS-K became weaker and insignificant as nutritional status worsened. However, the association between the MNA and SGDS-K scores was significant regardless of PSQI.

**Conclusion:**

Both sleep disturbance and malnutrition were significantly associated with late-life depression, although malnutrition may be more critically associated with depression than sleep disturbance in community-dwelling older adults.

## Introduction

Progression of population aging has promoted an increased emphasis on the negative consequences of late-life depression, which has been associated with functional impairment, worsening of coexisting medical problems, and cognitive decline (1). However, depressive symptoms in older adults have often been overlooked (2). This may be due to age-related differences in the phenomenology of depression (2, 3). Older adults with depression tend to experience more somatic symptoms than younger patients, which can easily be misinterpreted as symptoms of preexisting physical illness (3). Moreover, patients with late-life depression tend to be less recognitive and less expressive of affective symptoms owing to socio-cultural factors (3, 4). Hence, a significant number of patients encountered in the clinical field remain underdiagnosed and undertreated (2). Therefore, clinicians have been urged to pay careful attention to somatic presentations associated with late-life depression, especially sleep disturbance and malnutrition, which are the most frequent somatic presentations in patients with depression (5).

The prevalence of sleep disturbance in later adulthood is estimated to range from 40 to 70% and is known to increase with age (6, 7). Older adults frequently have trouble falling asleep as well as maintaining sleep, both of which are accompanied by changes in sleep architecture (8). Previous studies have revealed that sleep disturbances are more frequent in older adults with depression than in those without (9, 10). However, recent evidence also suggests that there is a bidirectional relationship between sleep and depression (11). Both sleep disturbance related to the occurrence or relapse of late-life depression and persistent sleep disturbance have been associated with non-remission (12). Therefore, researchers have proposed that treatment of sleep disorders may aid in alleviating depressive symptoms (11, 12).

Malnutrition is also an important clinical condition associated with late-life depression. Malnutrition is defined as inadequate nutritional status characterized by insufficient dietary intake, poor appetite, muscle wasting, and weight loss (13). The prevalence of malnutrition in older adults differs greatly based on the study setting (14). In Europe and North America, reported rates have ranged from 15% in non-institutionalized older adults to 65% in inpatient older adults (15). Depression is a well-known risk factor for malnutrition in older adults (15), and recent studies in nutritional psychiatry suggest a mutual relationship between malnutrition and depression mediated by various pathways, including those involved in inflammation (16). Consistent with this hypothesis, some researchers have promoted nutritional interventions for attenuating depression in older adults (17, 18).

Understanding the interrelationships among sleep, nutritional status, and depression in later adulthood is of particular importance in the context of geriatric medicine, in which depression among older adults may present within the context of multiple age-related disorders. This phenomenon, referred to as geriatric syndrome (19), is defined as a collection of comorbid clinical conditions among older adults that stem from declines in homeostatic reserve capacity (20, 21). In clinical practice, such conditions include frailty, cognitive decline, incontinence, sleep disturbance, malnutrition, and depression (20, 22). Given the complexity and multifactorial features of geriatric syndrome, understanding the interactions among these conditions is important for planning appropriate preventive strategies (21). Previous studies have investigated the interrelationships of sleep disturbance, malnutrition, and depression with other manifestations of geriatric syndrome (23–25); however, little research has focused on the interaction between sleep disturbance and malnutrition in the context of late-life depression.

The objective of this study was to examine the effects of sleep disturbance and malnutrition on depression among community-dwelling older adults. The authors hypothesized that both sleep disturbance and malnutrition exhibit significant associations with late-life depression and that these conditions interact to influence depressive symptomatology.

## Materials and Methods

### Participants

From January 2017 to April 2021, 1,029 community-dwelling older adults, aged between 60 and 90 years, who came to Suwon Geriatric Mental Health Center in the Republic of Korea for general screening of cognitive impairments, depression, and other possible mental health problems were analyzed for this study. The exclusion criteria were as follows: severe cognitive impairment based on a global deterioration score > 3 points or a Mini-Mental State Examination score < 18 points; history of another psychiatric disorder (schizophrenia, bipolar disorder, and alcohol use disorder); history of neurological or medical disorders, such as traumatic brain injury, brain tumor, and hemiparalysis; and participants who had missing values on independent, dependent, and covariates. This study was approved by the Institutional Review Board of Ajou University Hospital (AJIRB-SBR-SUR-16-122). All participants provided written informed consent.

### Assessment and Measurements

#### Sleep Disturbance

The Korean version of the Pittsburgh Sleep Quality Index (PSQI) was used to examine sleep disturbance. The PSQI is a widely used and validated tool for evaluating sleep quality. The PSQI consists of a self-report questionnaire on sleep quality during the past month. The PSQI has seven sub-scores, each with a score of up to three points (26). The total score ranges from 0 to 21, and a score of 9 or more is considered as problemed sleep (27).

#### Malnutrition

The Korean version of the MNA was used to assess nutritional status. The MNA is a well-validated test to measure the nutritional status of older adults, and it requires a short time to complete. The MNA consists of the following four groups of questions: anthropometric measurements, global assessment, dietary questionnaire, and subjective assessment (28). The MNA score ranges from 0 to 30, and older adults can be classified according to the total MNA score (MNA > 23.5, well-nourished; 17–23.5, risk of malnutrition; and MNA < 17; malnutrition) (29).

#### Depression

The Geriatric Depression Scale (GDS) consists of a self-reported yes–no questionnaire, and it is widely used in assessing depression in older adults due to simplicity. GDS has also been shown to be a useful screening tool for depressive symptoms in patients with mild cognitive impairment (30). The short form of the GDS (SGDS) consists of 15 questions (31). The Korean version of the GDS (GDS-K) was validated and The Korean version of the SGDS (SGDS-K) was validated as an adequate substitute for the GDS-K (32). In this study, the SGDS-K was used to assess depressive symptoms, with scores of 8 or higher considered indicative of depression (32).

#### Covariates

Variables known to be associated with depression, sleep, and nutrition were included in the analysis. Sociodemographic parameters, including age, sex, education, living status, and body mass index, were recorded (33). The link between physical illness and depression has been identified in many studies (34). Participants were asked regarding the presence of physical illness, including hypertension, diabetes mellitus, and cardiovascular diseases. Cardiovascular diseases include hyperlipidemia, heart failure, angina, myocardial infarction, and stroke.

### Statistical Analysis

To calculate the minimum required number of samples, G*power 3.1.9.7 software was used. Assuming a significance level of 0.05, small effect size, power of 0.8, and number of variables as 10, the analysis indicated that the minimum number of participants required was 822; thus, an adequate number of study participants were included (35). Numeric variables are expressed as means and standard deviations, and categorical variables are expressed as numbers and percentages. A multivariate linear regression analysis was conducted to examine the relationship between variables and SGDS-K scores, which represent the severity of depression in participants. In Model 1, only covariates including sociodemographic parameters and the presence of illness were included. Model 2 incorporated malnutrition represented by the MNA score and sleep disturbance represented by the PSQI score, in addition to the variables included in Model 1. To measure the local effect size of MNA and PSQI scores within Model 2, Cohen’s f^2^ of each variable was calculated, and f^2^ ≥ 0.02, f^2^ ≥ 0.15, and f^2^ ≥ 0.35 were interpreted as small, medium, and large effect sizes, respectively (36). In Model 3, the MNA and PSQI scores were centered, and the product term was added to Model 2 to evaluate interactions. We applied the Johnson–Neyman technique and generated the Johnson–Neyman plot to probe and visualize the conditional effect of MNA according to the change in PSQI and vice versa (37, 38). *P*-values less than 0.05 were considered statistically significant. All the analyses were performed using SPSS, version 25 (IBM, Chicago, IL, United States) or the R Statistical Software, version 4.1.0 (R Foundation for Statistical Computing, Vienna, Austria).

## Results

### General Characteristics of the Participants

Demographic characteristics of the participants are presented in Table 1. Among the 1,029 participants, 71.8% were female, and 28.2% were male. The mean age of the participants was 73.79 ± 6.92 years, and the average years of education was 6.58 ± 4.56 years. Of the participants, 59% lived alone. Regarding underlying disease, 55.4% had hypertension, 24.9% had diabetes, and 39.2% had cardiovascular disease. The average BMI of the study participants was 23.56 ± 3.39.

**TABLE 1:** Demographic characteristics of the study participants.

Variables^a^	All participants (*n* = 1,029)
Age, year	73.79 ± 6.92
Female (%)	739 (71.8)
Education year	6.58 ± 4.56
Living alone (%)	607 (59.0)
Hypertension (%)	570 (55.4)
Diabetes (%)	256 (24.9)
Cardiovascular disease (%)	403 (39.2)
Body Mass Index	23.56 ± 3.39
Mini-mental state examination score	25.65 ± 3.03
Korean Pittsburgh sleep quality index	9.86 ± 4.15
Good sleeper	400 (38.9)
Poor sleeper	629 (61.1)
Korean mini nutritional assessment	19.90 ± 4.00
Well nourished	171 (16.6)
Risk of malnutrition	647 (62.9)
Malnutrition	211 (20.5)
Korean short form of geriatric depression scale	8.79 ± 4.52
Non-depressed	403 (39.2)
Depressed	626 (60.8)

*^a^Values represented as the mean ± SD and number (%) for categorical variables. SD, standard deviation.*

### Effects of Sleep Disturbance and Malnutrition on Late-Life Depression

**FIGURE 1:**
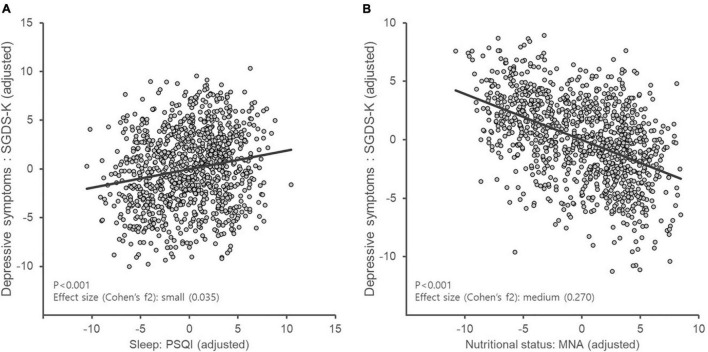
Partial regression plot of depression considering sleep disturbance and nutritional status. MNA, Mini Nutritional Assessment; PSQI, Pittsburgh Sleep Quality Index; SGDS-K, Korean version of the Short Form of Geriatric Depression Scale. According to Cohen’s guidelines, the local effect size was small for PSQI (Cohen’s f2: 0.035) **(A)** and medium for MNA (Cohen’s f2: 0.270) **(B)**.

The results of the multiple linear regression are shown in Table 2. In Model 1, sociodemographic parameters including age, sex, education, living status, and presence of physical illness including hypertension, diabetes, and cardiovascular disease were included. Model 1 was significant (adjusted *R*^2^ = 0.045 and *P* < 0.001), and age, sex, education, and living status were significantly associated with depression. While adjusting for all the same variables included and in Model 1, the MNA and PSQI scores were added to Model 2, which demonstrated a considerable increase in adjusted *R*^2^ value compared with Model 1 (adjusted *R*^2^ = 0.313 and *P* < 0.001). In Model 2, higher SGDS-K scores were significantly associated with lower MNA scores (standardized beta = −0.480, *P* < 0.001) and higher PSQI scores (standardized beta = 0.166, *P* < 0.001). Partial regression plots after adjusting for possible covariates are shown in Figure 1. According to Cohen’s guidelines, the local effect size was medium for MNA (Cohen’s f^2^: 0.270) and small for PSQI (Cohen’s f^2^: 0.035).

**TABLE 2:** Multiple linear regression analysis for associations of depression with sleep and nutrition.

	Dependent variable: SGDS-K score				
Independent variables	Unstandardized coefficient		Standardized coefficient		
Model 1 (covariates)	β	Std. error	β	Std. error	*P*-value
Age (years)	–0.071	0.021	–0.109	0.032	0.001
Sex	–0.887	0.324	–0.088	0.032	0.006
Education (years)	–0.163	0.033	–0.164	0.033	0.000
Living alone	1.269	0.282	0.138	0.031	0.000
Hypertension	0.013	0.295	0.001	0.032	0.966
Diabetes	0.421	0.328	0.040	0.031	0.199
Cardiovascular disease	0.296	0.290	0.032	0.031	0.307
BMI	–0.083	0.042	–0.062	0.032	0.050
**Model 2 (adjusted)**					
PSQI score	0.180	0.030	0.166	0.028	0.000
MNA score	–0.542	0.033	–0.480	0.029	0.000
**Model 3 (adjusted)**					
PSQI score * MNA score	0.014	0.007	0.051	0.026	0.048

*In Model 1, only covariates including sociodemographic parameters and the presence of illness were included. In Model 2, the PSQI and MNA scores were added to Model 1. In Model 3, the product terms of the PSQI and MNA scores were added to Model 2. BMI, body mass index; PSQI, Pittsburgh Sleep Quality Index; MNA, Mini Nutritional Assessment.*

### Interaction and Conditional Effect of Sleep Disturbance and Malnutrition on Late-Life Depression

**FIGURE 2:**
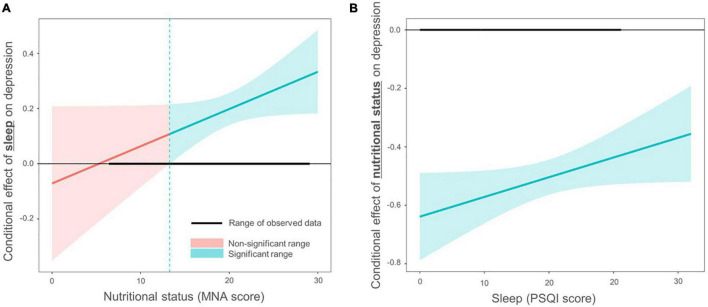
Interaction effects between sleep disturbance and nutritional status in older adults with depression. The Johnson–Neyman technique was employed to examine the interaction effect between sleep disturbance and nutritional status on depression. The y-axis represents the conditional effect on depression. Confidence intervals were displayed in different colors depending on whether they were significant (*P* < 0.05). **(A)** The PSQI score had an insignificant effect on SGDS-K when the MNA score was lower than 14.028, as shown in red. The range of the observed data is shown as a thick horizontal line. **(B)** The MNA score had a significant effect on SGDS-K regardless of the PSQI score, as shown in blue (n.s., non-significant). MNA, Mini Nutritional Assessment; PSQI, Pittsburgh Sleep Quality Index; SGDS-K, Korean version of the Short Form of Geriatric Depression Scale.

In Model 3, the product terms of MNA and PSQI were added to Model 2 to evaluate interactions. An omnibus *F*-test suggested a significant interaction effect of MNA and PSQI scores [*F*(11, 1017) = 43.919, *P* < 0.001]. The conditional effect of MNA was significant at the mean PSQI value (estimates = −0.543, 95% CI [-0.607, -0.479]). The conditional effect of PSQI was also significant at the mean MNA value (estimates = 0.181, 95% CI [0.122, 0.239]). To assess and visualize the conditional effects of sleep disturbance and malnutrition on late-life depression, the authors applied the Johnson–Neyman technique. The analysis indicated that the association between sleep disturbance and depression became weaker as nutritional status worsened, even becoming non-significant at a certain level (MNA < 14.028). However, the association between nutritional status and depression was significant regardless of the severity of sleep disturbance (Figure 2).

## Discussion

The current study examined the effects of sleep disturbance and malnutrition on depression in community-dwelling older adults. The results in this study demonstrated that both sleep disturbance and malnutrition were significantly associated with depression after adjusting for sociodemographic covariates. The local effect size was medium for malnutrition and small for sleep disturbance. Additionally, the interaction and conditional effects of sleep disturbance and malnutrition were measured. Sleep disturbance and malnutrition showed significant interaction. Moreover, the association between sleep disturbance and depression became insignificant in severe malnutrition while the association between malnutrition and depression was maintained in severe sleep disturbance.

Several studies have investigated the effects of sleep disturbance and malnutrition on depression. A meta-analysis demonstrated that greater sleep disturbance is associated with more severe depressive symptoms in older adults (10). Previous studies from different countries have also reported that late-life depression is associated with a risk of malnutrition (39, 40). The present results are consistent with these findings. However, no previous studies have considered sleep disturbance and malnutrition together. In the current study, sleep disturbance and malnutrition exerted a significant effect on late-life depression even in analyses considering each factor. The current results also indicated that the local effect size was larger for malnutrition than for sleep disturbance. Planning a balanced diet and cooking to ensure a healthy diet requires considerable effort for older adults (41), although lack of knowledge regarding a healthy diet has been cited as a potential cause of malnutrition among older adults (42, 43). Economic difficulties may also make healthy eating difficult (44). Moreover, for the older adult population, nutritional status may be an indicator of the quality of social support systems and social interactions. This association between social relationships and nutritional deficiency has also been confirmed in previous studies (45–47). Psychological factors and the social meaning of eating can also affect dietary patterns, including the type and amount of food intake (48). The existence of a social support system that can help overcome these challenges may help improve nutritional status in older adults.

Another interesting finding of this study is the interaction between sleep disturbance and malnutrition. After adjusting for sociodemographic variables and other physical conditions, the interaction between sleep disturbance and malnutrition was statistically significant. The inflammation hypothesis of late-life depression may explain this result. It considers age-related and comorbidity-related immune dysregulation as the etiology of late-life depression. Sleep deficiency results in the release of inflammatory markers, such as C-reactive protein and interleukin, which are associated with depression (11, 49). A relationship between malnutrition and inflammation has also been reported among older patients with physical illness (50), and research has indicated that patients with more severe malnutrition and higher inflammation experience more depressive symptoms (51). Moreover, inflammatory dietary pattern showed greater risk of developing depression (16). This evidence suggests that the inflammatory response may be key to understanding this interaction.

In addition, the conditional effect of sleep disturbance and malnutrition implies a priority between them. The association between sleep disturbance and depression became weaker in the malnourished state. These results emphasize the need for proper nutritional intervention programs in late-life depression. In previous studies, supplementation with micronutrients, such as vitamin B, vitamin D, and omega-3, has been shown to improve symptoms of depression in older adults (52–54). Another study has reported that a Mediterranean-based diet was associated with a lower risk of depression (18). However, the characteristics of older adults suggest the need for the development of tailored programs and cooperation of various occupations, including social staff (17, 41, 55). The findings in this study support the claims of previous studies that emphasize the importance of nutrition and suggest that multidomain intervention for older adults with depression should include nutritional evaluation and intervention.

There are some limitations to this study. First, as this was a cross-sectional study, it is difficult to determine the causal relationships among depression, sleep disturbance, and malnutrition. Further longitudinal studies are required to verify the current results. Second, the results may not be generalizable to the entire population given that participants were recruited from a mental health center, implying that the rate of coexisting psychiatric or physical illnesses may have been higher. For instance, the prevalence of depression in this study was higher than that reported in a previous nationwide study of older adults in Korea (56). However, the prevalence of depression can vary depending on the study setting (56), and another study conducted with patients treated at a Finnish community mental health care center reported results similar to ours (57). This discrepancy may indicate a need for specialized community mental health care centers that can screen for depression in older adults. The prevalence of sleep disturbance (58, 59) and malnutrition were also higher in the current study than in previous studies (60, 61). This may be related to the high prevalence of depression in the current study. Although the authors attempted to exclude participants with cognitive impairment, it is also possible that participants with mild cognitive decline were included. These issues and potential selection biases may limit the ability to generalize the results of this study, highlighting the need for large-scale, population-based studies in the future. Nevertheless, this study is meaningful since it explored the effects of two common conditions in geriatric syndrome on depression in community-dwelling older adults together and examined interactions between them, thereby suggesting the importance of nutritional intervention, which is often underestimated and overlooked in clinical practice. Third, few items in the MNA questionnaire may imply current depressed state of the participants and result in circular association. We performed an additional analysis using the MNA score except the items that matter. A significant relationship of sleep disturbance (standardized beta = 0.206, *P* < 0.001) and malnutrition (standardized beta = −0.393, *P* < 0.001) with depression was observed.

## Conclusion

In conclusion, the current results demonstrated that both sleep disturbance and malnutrition were significantly associated with late-life depression. A comparison of effect sizes indicated that malnutrition exerted a greater effect than sleep disturbance, although the interaction between the two was significant. In particular, the effect of sleep disturbance on depression was not significant in patients with severe malnutrition. This study highlights the potential importance of nutritional interventions when developing treatment strategies for community-dwelling older adults with depression.

## Data Availability Statement

The raw data supporting the conclusions of this article will be made available by the authors, without undue reservation.

## Ethics Statement

This study was approved by the Institutional Review Board of Ajou University Hospital (AJIRB-SBR-SUR-16-122). The patients/participants provided their written informed consent to participate in this study.

## Author Contributions

GH: conceptualization, writing—original draft, and formal analysis. CH and SS: writing—review, editing, methodology, funding acquisition, and supervision. HR: conceptualization, methodology, investigation, writing—original draft, and formal analysis. All authors have approved the final article.

## Conflict of Interest

The authors declare that the research was conducted in the absence of any commercial or financial relationships that could be construed as a potential conflict of interest.

## Publisher’s Note

All claims expressed in this article are solely those of the authors and do not necessarily represent those of their affiliated organizations, or those of the publisher, the editors and the reviewers. Any product that may be evaluated in this article, or claim that may be made by its manufacturer, is not guaranteed or endorsed by the publisher.
